# 3D‐Bioprinted Osteoblast‐Laden Nanocomposite Hydrogel Constructs with Induced Microenvironments Promote Cell Viability, Differentiation, and Osteogenesis both In Vitro and In Vivo

**DOI:** 10.1002/advs.201700550

**Published:** 2017-11-24

**Authors:** Xinyun Zhai, Changshun Ruan, Yufei Ma, Delin Cheng, Mingming Wu, Wenguang Liu, Xiaoli Zhao, Haobo Pan, William Weijia Lu

**Affiliations:** ^1^ Research Center for Human Tissue and Organs Degeneration Institute Biomedical and Biotechnology Shenzhen Institutes of Advanced Technology Chinese Academy of Sciences Shenzhen 518055 China; ^2^ Department of Orthopaedic and Traumatology The University of Hong Kong 21 Sassoon Road Pokfulam Hong Kong 999077 China; ^3^ School of Materials Science and Engineering Tianjin Key Laboratory of Composite and Functional Materials Tianjin University Tianjin 300352 China

**Keywords:** 3D‐bioprinting, nanocomposite hydrogels, osteoblast‐laden constructs, osteogenesis capability

## Abstract

An osteoblast‐laden nanocomposite hydrogel construct, based on polyethylene glycol diacrylate (PEGDA)/laponite XLG nanoclay ([Mg_5.34_Li_0.66_Si_8_O_20_(OH)_4_]Na_0.66, clay_)/hyaluronic acid sodium salt (HA) bio‐inks, is developed by a two‐channel 3D bioprinting method. The novel biodegradable bio‐ink A, comprised of a poly(ethylene glycol) (PEG)–clay nanocomposite crosslinked hydrogel, is used to facilitate 3D‐bioprinting and enables the efficient delivery of oxygen and nutrients to growing cells. HA with encapsulated primary rat osteoblasts (ROBs) is applied as bio‐ink B with a view to improving cell viability, distribution uniformity, and deposition efficiency. The cell‐laden PEG–clay constructs not only encapsulated osteoblasts with more than 95% viability in the short term but also exhibited excellent osteogenic ability in the long term, due to the release of bioactive ions (magnesium ions, Mg^2+^ and silicon ions, Si^4+^), which induces the suitable microenvironment to promote the differentiation of the loaded exogenous ROBs, both in vitro and in vivo. This 3D‐bioprinting method holds much promise for bone tissue regeneration in terms of cell engraftment, survival, and ultimately long‐term function.

## 1 Introduction

Traditional strategies for bone tissue engineering are based on the facilitation of cell growth into engineered interconnecting scaffolds to generate a functional tissue construct for the reestablishment of structure and function in damaged bone tissues.^1^ However, the realization of the desired levels of cell deposition and cell distribution in 3D scaffolds remains a great challenge. Recent developments in the tempospatial specific 3D‐bioprinting of cells and inks show promise for a new approach in bone tissue engineering.^2^ With this technology, delicately tailored cell‐laden tissue constructs have been reportedly constructed to regenerate bone tissue. Zhang and co‐workers recently reported on bone mesenchymal stem cells (BMSCs)‐laden gelatin/sodium alginate/carboxymethyl chitosan hydrogel scaffolds prepared through a 3D‐bioprinting method. The prepared scaffolds exhibited good mechanical properties and favorable cytocompatibility, with cell viability of over 85% postprinting.^3^ Neufurth et al. encapsulated bone‐related SaOS‐2 cells into a biologically inert sodium alginate matrix and used a second layer containing polyP·Ca^2+^ to cover the bioprinted cell laden scaffold. The encapsulated cells exhibited a good proliferation rate and mineralization ability.^4^ These previous studies were mainly devoted to imitating the native structure of bone tissues and improving the viability of 3D‐bioprinted cells in the short term. However, whether or not the 3D‐bioprinted cells can realize the functional features of bone tissue after in vivo implantation still remains unknown. Indeed, the long‐term in vivo evaluation of most of the previously reported cell‐laden scaffolds for bone regeneration is deficient and limited, due to the lack of ideal bio‐inks for 3D‐bioprinting to favorably support cell growth and development both in the short and long terms.

In extrusion‐based printing, the materials which can be used as bio‐inks for bone tissue regeneration should satisfy the following basic requirements: (1) extrusion properties—suitable rheology for 3D‐bioprinting; (2) stability—the printed scaffolds should not collapse before solidification or crosslinking; (3) good biocompatibility and porosity—high cell viability after printing, with optimal avenues for the adequate diffusion of nutrients and oxygen to facilitate cell proliferation or differentiation in the short term; (4) appropriate mechanical properties; and (5) osteogenic capability to promote differentiation and new bone formation in the long term. Frequently used bio‐inks such as gelatin,^5^ alginate,^6^ gelatin/alginate,^4^ gelatin/alginate/chitosan,^3^ and poly(ethylene glycol) (PEG) dimethacrylate/gelatin^7^ that have been used in the fabrication of cell‐laden scaffolds have shown high biocompatibility for cell viability in bone repair. However, they lack osteogenic capability to promote cell differentiation and new bone formation in the absence of growth factors, which represents a clear limitation for their successful application. Pati et al. first attempted to develop novel decellularized extracellular matrix (dECM) bio‐inks which could provide an optimized microenvironment and was conducive to the growth of 3D structured tissue in the long term. Their potential application for adipose, cartilage, and heart tissue regeneration was explored, but bone tissue was not mentioned.^8^ Moreover, the inducement properties of this bio‐ink were only validated in vitro and the tedious preparation process and expensive cost further limit its application prospects.

We report herein on the development of a novel biodegradable bio‐ink comprised of polyethylene glycol diacrylate (PEGDA) and laponite XLG nanoclay, which was photo‐crosslinked to form a stable gel (PEG–Clay) to support the printing process, facilitate optimal cell growth and function through the delivery of nutrients and oxygen, and promote osteogenesis due to the induced microenvironment forming by the released magnesium ions (Mg^2+^) and silicon ions (Si^4+^). Simultaneously, another bio‐ink, hyaluronic acid sodium salt (HA) with encapsulated cells, was designed to improve cell viability, distribution uniformity, and deposition efficiency. The two bio‐inks, via a two‐channel 3D‐bioprinting method, were alternately extruded to fabricate primary rat osteoblast‐laden (ROB‐laden) nanocomposite hydrogel constructs for bone regeneration. We evaluated the proliferation and differentiation behavior of the loaded cells and the osteogenic capability of ROB‐laden nanocomposite hydrogel constructs both in vitro and in vivo.

## 2 Results

### 2.1 Characterization of Bio‐Inks A and B

**Figure 1:**
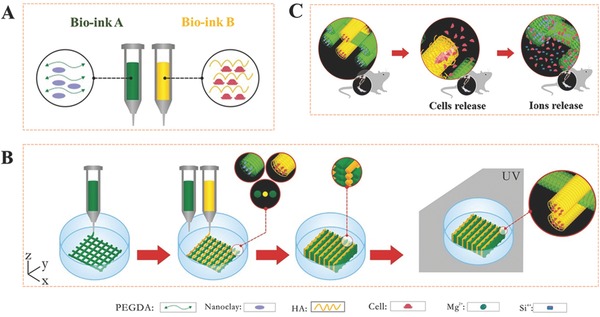
Stepwise illustration depicting the two‐channel bio‐ink system and method used for 3D‐bioprinting and the in vivo experiments. The composition of bio‐ink A and bio‐ink B (A), 3D‐bioprinting process of the ROB‐laden constructs using a two‐channel method (B) and the diagram for the in vivo experiments (C).

**Figure 2:**
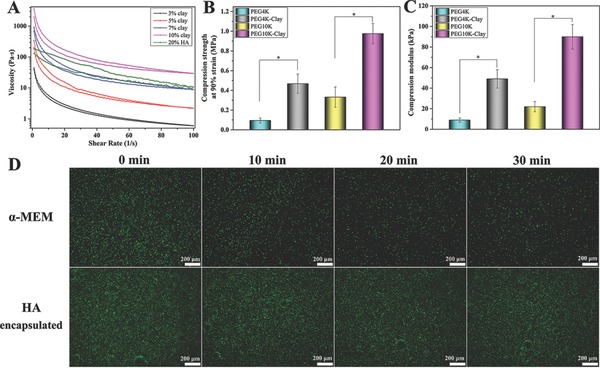
Rheology characteristics of 20%PEG4K–Clay prehydrogel solutions with different nanoclay concentrations and the 20% HA solution A). Compression strength B) and compression modulus C) of PEG hydrogels and PEG–Clay hydrogels. The effect of UV exposure on ROBs in α‐MEM medium and encapsulated in 20% HA solution D). Asterisks (*) denote significant differences (*p < 0.05).

The 3D‐bioprinting of the cell‐laden constructs using two bio‐inks was performed over a few steps (**Figure** **1**). The two bio‐inks, denoted bio‐ink A and bio‐ink B, were prepared first. A PEG–Clay prehydrogel solution was designed for bio‐ink A containing photo‐crosslinkable PEGDA and the nanoclay, while HA was used to encapsulate cells as bio‐ink B. We synthesized biodegradable photo‐crosslinkable PEGDA with two classical molecular weights, *M*_w_ = 4K and 10K, using acryloyl chloride according to a method previously reported.^9^ ^1^H NMR spectra (Figure S1, Supporting Information) confirmed the successful formation of PEGDA4K and PEGDA10K. Then, the nanoclay was added to a 20% PEGDA (w/v) aqueous solution, resulting in a thickening of the viscosity from liquid form to sticky, rendering it suitable for room temperature 3D‐bioprinting (**Figure** **2**A). As no obvious difference could be observed between the viscosities of the PEGDA4K and PEGDA10K solutions at 20% concentration, rheological analysis of 20%PEG4K–Clay prehydrogels with different nanoclay concentrations was performed. When less than 7% nanoclay (w/v) was added to the 20% PEGDA solution, the printed scaffold could not maintain a fixed shape with fused microstructure. When the nanoclay concentration exceeded 7%, the viscosity of the PEG–Clay prehydrogel solution was too high to be printed under lower pressure at room temperature. Moreover, the viscosity of the 20%PEG–7%Clay prehydrogel solution exhibited similar rheological behavior to 20% HA (w/v). Therefore, 7% nanoclay was selected as the optimal concentration to add to the 20% PEGDA aqueous solutions. UV light exposure led to the gelation of the PEG–Clay prehydrogel solution and Fourier transform infrared (FTIR) spectra confirmed the successful formation of the PEG–Clay hydrogels (Figure S2, Supporting Information). Thermogravimetric analysis (TGA) (Figure S3, Supporting Information) indicated that approximately 25% nanoclay was incorporated into the PEG–Clay hydrogels, in both 20%PEG4K–7%Clay and 20%PEG10K–7%Clay, which is very close to the original clay concentration (26%, relative to the total mass of PEGDA and nanoclay), and further confirmed the successful incorporation of nanoclay into the hydrogel matrix. Transmission electron microscopy (TEM) images highlight the good exfoliation and dispersion of nanoclay within the PEG4K–Clay hydrogel, with no obvious agglomeration observed, Figure S4 in the Supporting Information. The well dispersed nanoclay deposits contribute to the attractive mechanical properties of the PEG–Clay hydrogels over pure PEG hydrogels. As presented in Figure 2B,C, the compression strength values recorded for the pure 20%PEG4K and 20%PEG10K hydrogels were 0.096 and 0.332 MPa, respectively. The compression strength increased upon incorporation of the nanoclay, with values of 0.468 and 0.976 MPa recorded for the 20%PEG4K–7%Clay and 20%PEG10K–7%Clay hydrogels, respectively. Moreover, the compression modulus increased from 9 and 49 to 22 and 90 kPa for the pure 4K and 10K PEG hydrogels and their nanoclay incorporating cousins, respectively. These results confirm that adding nanoclay to the hydrogel systems not only imparted printability to the PEG–Clay prehydrogel solutions but also enhanced the mechanical properties of the hydrogels, thus increasing the potential of the PEG–Clay hydrogels as component supporting materials for bone tissue regeneration.

HA, a highly biocompatible material, was used without further crosslinking as a sacrificial material, to facilitate the gradual and uniform release of encapsulated cells within the scaffold 24 h after printing. Furthermore, the HA layer protects cells from UV damage during the PEG–Clay hydrogel crosslinking procedure. Aqueous 20% HA is similar in viscosity to the 20%PEG–7%Clay prehydrogel solution (Figure 2A) and was suitable for the 3D‐bioprinting of cells. The choice of concentration is important, not only for printability but also the primary hydrogel structure should not be impacted by the addition of the second material.^10^ Moreover, when the HA concentration exceeds 20%, the printing pressure needed for successful printing would surpass 100 kPa, which would negatively impact on the viability of the printed cells.^8^, ^11^ Due to the susceptibility of cells to UV‐related damage,^12^ we evaluated the ability of HA to act as a protective barrier against UV‐damage (Figure 2D). ROBs directly dispensed in alpha minimum essential medium (α‐MEM) medium were acutely damaged by UV‐irradiation over time. However, when encapsulated in 20% HA and exposed to UV‐irradiation, no obvious damage was observed, even when the irradiation time was as long as 30 min. These results indicate that bio‐ink B (HA) was an ideal carrier for loading cells within the scaffold.

To summarize, 20%PEG–7%Clay and 20% HA encapsulated cells were chosen as bio‐ink A and bio‐ink B, respectively, to construct cell‐laden scaffolds for further investigation in this study.

### 2.2 3D‐Bioprinting of ROB‐Laden Constructs Using a Two‐Channel Method

**Figure 3:**
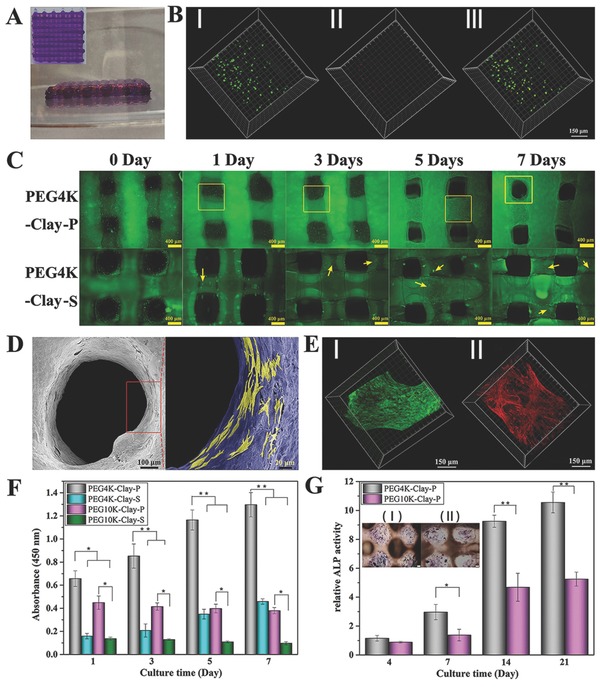
The final structure of 3D‐bioprinted scaffolds prepared by the two‐channel method, PEG–Clay was stained with gentian violet and HA was stained with Rhodamine A). Live/dead assay of ROBs 1 d after printing within PEG4K–Clay scaffolds B), I) viable ROBs (green, calcein AM), II) dead ROBs (red, EthD‐1), and III) merged. The status of ROBs after 3D‐bioprinting (PEG4K–Clay‐P) or traditional seeding (PEG4K–Clay‐S) on PEG4K–Clay scaffolds after culturing for 0, 1, 3, 5, and 7 d C). SEM images of ROBs 3D‐bioprinted within PEG4K–Clay scaffolds after 7 d of culture (D, ROBs in the red rectangular were marked with yellow color). Live/dead assay E, I) and cytoskeleton staining E, II) of ROBs 3D‐bioprinted within PEG4K–Clay scaffolds after 7 d of culture. Cell counting Kit‐8 (CCK‐8) analysis of ROBs after 3D‐bioprinting within PEG–Clay scaffolds (PEG–Clay‐P) or traditionally seeded on PEG–Clay scaffolds (PEG–Clay‐S) on days 1, 3, 5, and 7 d of culture F). ALP analysis of ROBs 3D‐bioprinted within PEG–Clay scaffolds after culturing for 4, 7, 14, and 21 d G), the insert images show ALP staining of ROBs on PEG4K–Clay‐P I) and PEG10K–Clay‐P II). Asterisks (*) denote significant differences (*p < 0.05, **p < 0.01).

The ROB‐laden constructs were layer by layer fabricated through a two‐channel method, as depicted in Figure 1. The first layer as a stable base was built with only bio‐ink A. From the second layer to the end layer, bio‐ink A and bio‐ink B were alternately extruded to build a cell‐laden construct. Movie S1 in the Supporting Information shows the actual printing process and **Figure** **3**A shows the final structure of the scaffold. As the PEG–Clay prehydrogel solution could maintain its structure at room temperature, it was possible to first print the scaffolds individually and then photopolymerize them together. As photo‐crosslinking time increases, the degree of PEG–Clay scaffold crosslinking naturally increases.^13^ In order to minimize possible damage and maintain the viability of the ROBs and ensure the formation of the PEG–Clay hydrogels in our constructs, 10 min UV‐irradiation was chosen.

After UV‐irradiation, 3D‐bioprinted nanocomposite hydrogel constructs with well‐distributed cells for bone regeneration were formed. As shown in Figure 3C, when cells are seeded on pure 3D‐printed PEG–Clay scaffolds by the traditional cell‐seeding method, even if enough time is given for cell adhesion, most of the cells drop through the holes of the scaffold or just adhere to the surface, and ultimately are distributed nonuniformly.^14^ In contrast, the number and distribution of cells within a scaffold can be precisely regulated in the proposed 3D‐bioprinting system. After one‐day of culture, most of the cells adhered within the scaffolds and the viability of the printed cells was higher than 95% (Figure 3B). The results show that, our 3D‐bioprinted nanocomposite hydrogel constructs resulted in uniform cell distribution, effective deposition, and excellent viability.

### 2.3 In Vitro Proliferation and Cell Morphology

We further evaluated and compared the cell proliferation ability of PEG–Clay cell matrices prepared by both the 3D‐bioprinting method and the traditional cell‐seeding method (Figure 3C,F). Regardless of the cell seeding protocol, the proliferation of ROBs on the PEG4K–Clay scaffolds was better than the PEG10K–Clay scaffolds. In the PEG10K–Clay scaffolds, cells maintained their spherical morphology and poorly spread, and while the cells were still alive they did not proliferate well. For the PEG4K–Clay scaffolds, the highest rate of proliferation was observed on days 3–5 of culture, in both the cell printing and traditional seeding methods. In the traditional cell‐seeding approach, only a few cells had adhered and spread out on the PEG4K–Clay scaffolds after 1 d of culture, and even after 3 d no obvious cell–cell contact could be observed. Due to the nonuniform distribution of cells, single cells and cell aggregation could be observed after 7 d of culture (Figure 3C and Figure S5, Supporting Information). In contrast, for the 3D‐bioprinted cells, though few cells retained their spherical morphology after 3 d of culture, the cells were well distributed and spread after 5 d (Figure S5, Supporting Information). After 7 d of culture, almost complete ROBs coverage of the scaffolds was realized with almost no dead cells observed, as visualized by F‐actin staining of the cytoskeleton and live/dead experiment (Figure 3E). Furthermore, since the cell‐containing component (HA) was printed between each of the PEG–Clay prehydrogel lines, the cells not only grew on the surface of the matrices but also grow within the scaffolds themselves, as confirmed by scanning electron microscope (SEM) imaging (Figure 3D and Figure S5, Supporting Information).

### 2.4 Osteogenic Differentiation of ROBs after 3D‐Bioprinting

Alkaline phosphatase (ALP) activity assays and ALP staining were used to evaluate printed ROBs differentiation after 4, 7, 14, and 21 d of culture (Figure 3G). After printing within both PEG4K–Clay and PEG10K–Clay scaffolds, the cells always demonstrated ALP secretion ability, with the largest increase observed at 14 d. The ALP activity of ROBs within the PEG4K–Clay scaffolds was significantly higher than within the PEG10K–Clay scaffolds on days 7, 14, and 21 d of culture. ALP staining was used to confirm these results after 21 d of culture (Figure 3G). The images show that the ROBs within the PEG10K–Clay scaffolds were almost aggregated compared with the well spread conformation of cells growing within the PEG4K–Clay scaffolds. Moreover, the bluish intensity, which is directly proportional to the ALP activity, was much higher for the PEG4K–Clay scaffolds than the PEG10K–Clay scaffolds, which is consistent with the cell proliferation and differentiation results outlined.

This phenomenon can partly be attributed to the incorporation of nanoclay into our 3D‐bioprinting systems; as PEG hydrogels have no osteogenic properties,^15^ cells cannot grow or spread out on pure PEG hydrogels regardless of the PEG molecular weight.^16^ The mechanical properties of PEG alone are too weak for cells to adhere, by dispersing nanoclay within the system the mechanical properties are significantly improved upon, as described (Figure 2B,C). Moreover, our PEG–Clay scaffolds can gradually release bioactive ions (magnesium ions, Mg^2+^ and silicon ions, Si^4+^), which act to promote osteogenic differentiation.^17^ Figure S6 in the Supporting Information presents the release curve of Mg^2+^ and Si^4+^ from PEG–Clay scaffolds. At all time points, the ion concentrations released from the PEG10K–Clay scaffolds were marginally higher than the PEG4K–Clay scaffolds, probably due to the lower crosslinking density of the PEG10K–Clay scaffolds. The measured ion concentrations were in the range considered effective for eliciting bone regeneration.^18^, ^19^ The release concentration increased steadily for both sample types, from 44 and 56 µg mL^−1^ after 1 d of immersion to 106 and 115 µg mL^−1^ after 21 d of immersion, for Mg^2+^ released from PEG4K–Clay and PEG10K–Clay scaffolds, respectively. The release concentration of Si^4+^ increased from 108 and 128 µg mL^−1^ after 1 d of immersion to 245 and 263 µg mL^−1^ after 21 d of immersion, for PEG4K–Clay and PEG10K–Clay scaffolds, respectively. Notably, after 7 d of immersion, the release rate reduced, most likely because the surface ions are released more easily and faster than ions inside of the scaffolds, which are restricted and slow to release due to the slow degradation rate of the PEG‐based hydrogel.^20^

### 2.5 In Vivo Tibia Repair Experiments

**Figure 4:**
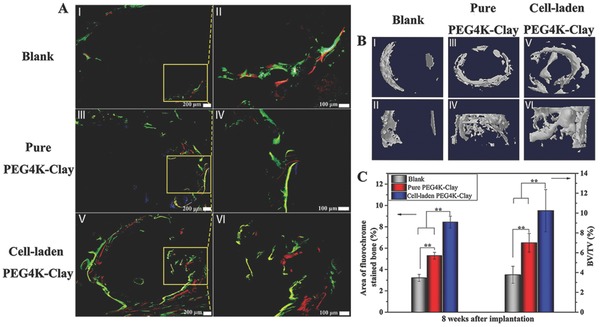
Sequential fluorescent labeling of blank, PEG4K–Clay scaffolds without ROBs, and PEG4K–Clay scaffolds with 3D‐bioprinted ROBs A). Yellow, red, and green represent tetracycline hydrochloride, alizarin red S, and calcein labeling, respectively. Characterization of implants and new bone formation by reconstructed 3D models after micro‐CT analysis B). Percentage of the area of fluorochromes‐stained bone and new bone volume/total volume (BV/TV) ratio C) (*p < 0.05, **p < 0.01, blank as a control).

Taking into account the superior performance of the 3D‐bioprinted ROB‐laden PEG4K–Clay scaffolds in the in vitro studies over their higher molecular weight cousins, they and pure PEG4K–Clay scaffolds were assessed in tibia repair and ectopic osteoinduction experiments in vivo. Sequential fluorescent labeling^21^ was used to monitor new bone formation around the injuries by applying three types of fluorochromes (**Figure** **4**A): tetracycline (yellow) for 2–4 weeks, alizarin red (red) for 4–6 weeks and calcein (green) for 6–8 weeks. Since fluorochrome labels can bind calcium ions of newly formed bone and get incorporated into the site of mineralization, the new bone formed at different periods can be discriminated by different fluorochrome labels. Comparing the pure PEG4K–Clay scaffolds and the blank group with the ROB‐laden PEG4K–Clay scaffolds, more continuous and more abundant fluorescent emission was observed for the 3D‐bioprinted PEG4K–Clay scaffolds. The percentage area occupied by the three fluorochromes in the labeled bone was calculated and is shown graphically in Figure 4C. The fluorochrome area of the ROB‐laden PEG4K–Clay scaffold was 8.44%, clearly larger than the equivalent areas of the pure PEG4K–Clay scaffold (5.30%) and the blank group (3.22%). Furthermore, different from all three colors can be observed for both the pure PEG4K–Clay scaffolds and the ROB‐laden PEG4K–Clay scaffolds, the yellow color can hardly be observed in the blank group, which means new bone formed very slowly without promotion. Micro–computed tomography (Micro‐CT) 3D‐images showed that the highest amount of new bone was found in the marrow cavity around or inside the ROB‐laden PEG4K–Clay scaffolds (Figure 4B). The bone volume of the ROB‐laden PEG4K–Clay scaffolds (10.24%) was significantly larger than that of the pure PEG4K–Clay scaffolds (7.20%) and the blank group (3.78%; Figure 4C).

**Figure 5:**
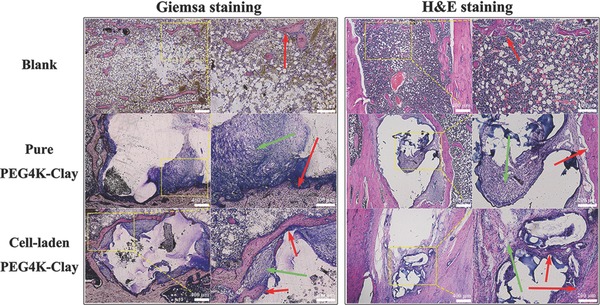
Histological staining observations of the whole bone around the defects with low magnification (40×) and high magnification (100×), marked with the yellow rectangle. Giemsa staining and H&E staining were used to stain new bone tissue in the blank group, pure PEG4K–Clay group, and the cell‐laden PEG4K–Clay group. The red arrows show the formation of new bone and the green arrows show the fibrous tissues around the scaffolds 8 weeks after surgery.

As shown in **Figure** **5**, the histological sections (8 weeks postimplantation), processed by Giemsa staining,^18^ gave a full view of bone formation in the three groups. The new bone formed (pink part) around the defect area in the blank group was intermittent. The newly formed bone in both of the PEG4K–Clay groups was far more continuous, especially in the ROB‐laden PEG4K–Clay scaffolds. For pure PEG4K–Clay scaffolds, although new bone can only be observed around the scaffolds, osteoblasts and fibroblasts (purple and blue) can be found within the scaffolds, which means that cells had grown into the scaffold and indicates that new bone may form within the scaffold later. While osteoblasts and fibroblasts can be observed within the ROB‐laden PEG4K–Clay scaffolds, new bone can also be clearly observed around and within the scaffolds. H&E staining^22^, ^23^ was used to further validate the Giemsa staining results (Figure 5). The results were consistent with the data collected from micro‐CT and new bone labeling analyses, verifying that PEG4K–Clay nanocomposite hydrogel scaffolds can stimulate new bone formation, and that the loading of exogenous cells into the scaffolds through 3D‐bioprinting has further positive implications for bone regeneration.

### 2.6 Ectopic Osteoinduction Experiments

**Figure 6:**
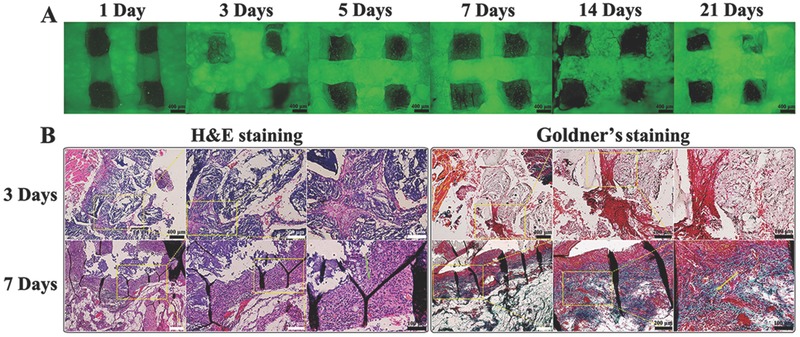
GFP‐labeled ROBs within 3D‐bioprinted PEG4K–Clay scaffolds after the in vivo ectopic osteoinduction experiment at 1, 3, 5, 7, 14, and 21 d time points A). H&E staining and Goldner's staining of 3D‐bioprinted PEG4K–Clay scaffolds after the in vivo ectopic osteoinduction experiment at 3 and 7 d under different magnifications (40×, 100×, and 200×) B). The green arrow shows the fibrous tissues and the yellow arrow shows the osteoid part.

**Figure 7:**
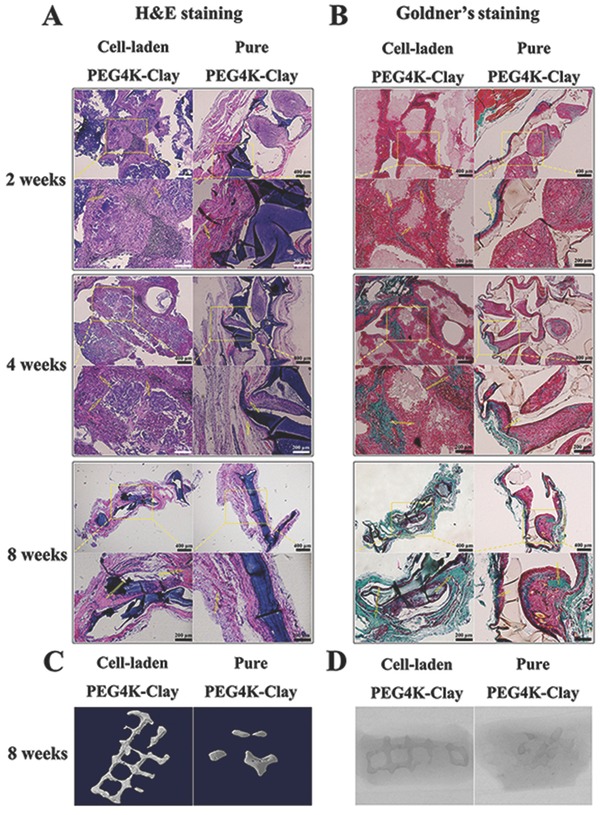
Histological staining observations of the in vivo ectopic osteoinduction experiment after implantation for 2, 4, and 8 weeks with low magnification (40×) and high magnification (100×), marked with the yellow rectangle. H&E staining A) and Goldner's staining B) were used to stain new bone tissue in the cell‐laden PEG4K–Clay group and pure PEG4K–Clay group. The yellow arrows show the formation of new bone after surgery. The reconstructed 3D models C) and micro‐CT picture D) were used to further confirm the formation of new bone 8 weeks after surgery.

To further investigate the growth behavior of printed allogenic ROBs after in vivo implantation, PEG4K–Clay printed with green fluorescent protein (GFP)‐labeled ROBs scaffolds (GROB‐laden PEG4K–Clay scaffolds) were implanted into the muscles of Sprague–Dawley rats (SD rats) for ectopic osteoinduction. After 1, 3, 5, 7, 14, and 21 d of implantation, the implanted GROB‐laden PEG4K–Clay scaffolds were removed from the muscles, and the fibrous tissue growing around the scaffolds was removed for the observation of cell status within the scaffolds using fluorescence microscopy. As time progressed, the implants increasingly integrated with the surrounding tissues, so that it became increasingly difficult for the scaffolds to be exfoliated. It is obvious from **Figure** **6**A that GFP‐labeled ROBs still persisted even 21 d after implantation, indicating that no obvious immunoreaction occurred after implantation. This result was supported by H&E and Goldner's staining,^24^ after GROB‐laden PEG4K–Clay scaffolds were implanted for 3 and 7 d (Figure 6B). For both the 3 and 7 d implantations, no significant immunoreaction was observed. Compared with the 3 d implants, the 7 d implant scaffolds were filled with fibrous tissue and even osteoids were identified. We further comparitively explored the osteogenic properties of both the ROB‐laden PEG4K–Clay scaffolds and the pure PEG4K–Clay scaffolds as the controls, through H&E and Goldner's staining (**Figure** **7**) after 2, 4, and 8 weeks of implantation. After the first 2 weeks, a small quantity of new bone had already formed in the ROB‐laden PEG4K–Clay scaffolds, while new bone in pure PEG4K–Clay scaffolds was not obvious. After 4 weeks of implantation, numerous osteoblast cells and new bone can be observed around and even inside the ROB‐laden PEG4K–Clay scaffolds, and the amount of new bone formed around the ROB‐laden PEG4K–Clay scaffolds was larger than that of pure PEG4K–Clay scaffolds. After 8 weeks of implantation, both new bones and numerous osteoblast cells can be observed around the ROB‐laden PEG4K–Clay scaffolds and the pure PEG4K–Clay scaffolds. However, both the new bone volume and thickness around the ROB‐laden PEG4K–Clay scaffolds were much larger than the pure PEG4K–Clay scaffolds. These results further confirm that our PEG–Clay nanocomposite hydrogels could promote new bone formation and that the ROB loaded 3D‐printed PEG–Clay scaffolds showed better osteogenic properties.

## 3 Discussion

In this study, ROB‐laden nanocomposite hydrogel constructs were fabricated by a two‐channel 3D‐bioprinting method by alternately extruding two bio‐inks (A and B). Bio‐ink A, PEG–Clay prehydrogel solution, offered a suitable viscosity for facilitating the process of 3D bioprinting, oxygen and nutrient delivery, and ultimately cell growth after crosslinking. The uniform distribution of the nanoclay in the PEG matrix not only enhanced the hydrogel mechanical properties but also rendered them more conducive to cell adhesion and proliferation than pure PEG hydrogels. Furthermore, the release of Mg^2+^ and Si^4+^ bioactive ions from the PEG–Clay scaffolds formed an induced microenvironment which stimulated the osteogenic differentiation of ROBs, to benefit bone regeneration. Simultaneously, bio‐ink B, based on HA, was applied as a vector to accurately and uniformly deposit an ROB load into the 3D‐printed scaffolds. The inclusion of HA not only guaranteed cell viability during 3D‐bioprinting with good distribution within the scaffolds, but its slow dissolution allowed for the gradual release of cells. Moreover, compared with the one‐channel method of 3D‐bioprinting, our two‐channel 3D‐bioprinting approach not only enhanced cell viability but also encouraged better cell spreading and proliferation.^25^

Importantly, this study also provides an efficient alternative to load cells within scaffolds for tissue engineering. Compared with the traditional cell‐seeded scaffolds, our ROB‐laden scaffolds showed better cell distribution and effective cell deposition in vitro. Although ROBs survived and grew on the PEG–Clay scaffolds whether traditionally seeded or printed, the distribution of cells in bioprinted scaffolds was much better than in the cell‐seeded scaffolds. In the seeded scaffolds, most of the cells dropped through the scaffold holes leading to nonuniform distribution, while in the ROB‐laden scaffolds, cell encapsulation in HA ensured initial uniform cell deposition followed by slow cell release to allow for optimal cell adhesion within the scaffolds. Even though the same cell numbers were used for both protocols, a significantly higher number of cells could be observed in the cell‐laden bio‐printed scaffolds. After 7 d of culture, ROBs almost entirely covered the bio‐printed scaffold surfaces, with practically no dead cells observed; only a few dispersed ROBs were found on the traditionally seeded scaffolds.

Moreover, the ROB‐laden PEG–Clay constructs also exhibited excellent osteogenic capability both in vitro and in vivo. In vitro experiments showed that ROBs successfully proliferated and differentiated, especially within the cell‐laden bio‐printed PEG4K–Clay scaffolds. For the tibia repair experiments, sequential fluorescent labeling results reveal the new bone formation process. Compared with the slow bone formation in the blank group, the new bone formation for both the pure PEG4K–Clay scaffolds and the ROB‐laden PEG4K–Clay scaffolds can be observed throughout the entire examination period, encouraged in‐part by the release of bioactive magnesium ions and silicon ions, which are well recognized promotion factors for osteogenic differentiation.^18^, ^19^ Furthermore, loading cells within PEG–Clay scaffolds by our 3D‐bioprinting system further improved the bone formation ability in vivo, suggesting that exogenous bone‐related cells can also play a key factor in the promotion of bone regeneration. Additionally, the favorable integration of exogenous cells was further highlighted during the in vivo ectopic osteoinduction study. The GFP‐labeled ROBs, from allogenic childhood SD rat calvarial chips, within PEG4K–Clay constructs were tracked after implantation in the muscles of adult SD rats for 21 d, and no significant immune response was observed in the surrounding tissues, which points to the potential of replacing bone‐related stem cells with exogenous allogenic osteoblasts for bone regeneration. Prolonging the implantation time, new bone formation in muscles was found in both pure PEG4K–Clay scaffolds and ROB‐laden PEG4K–Clay scaffolds. Additionally, the osteogenic capability of ROB‐laden PEG4K–Clay scaffolds was significantly better than that of pure PEG4K–Clay scaffolds. The 3D‐bioprinting cell loaded PEG–Clay system described herein holds significant promise for therapeutic application in bone regeneration, as evidenced by the in vitro and in vivo studies described, the results of which point to not only the excellent osteoblast scaffold distribution and viability in the short term but also the ability to promote new bone formation in the long term.

## 4 Conclusion

In summary, we successfully fabricated an osteoblast‐laden nanocomposite hydrogel construct via a two‐channel 3D‐bioprinting method. One channel carried bio‐ink A, PEG–Clay prehydrogel solution, which was suitably viscous to facilitate the 3D‐bioprinting process and was conducive to the delivery of oxygen and nutrients and cell growth after crosslinking. The other channel guided the accurate delivery of cells into the 3D scaffolds, using ROBs encapsulated in 20% HA solution. The HA component served to not only protect the ROBs from UV damage during the crosslinking process but also guaranteed uniform distribution and cell viability (more than 95% after 1 d). Furthermore, ROBs within the bioprinted scaffold showed better proliferation and differentiation than the same number of ROBs seeded on 3D PEG–Clay scaffolds. In tibia repair and ectopic osteoinduction experiments, ROB‐laden PEG–Clay scaffolds showed excellent osteogenic potential, due to the induced environment formed around the PEG–Clay scaffolds which was conducive to ROBs differentiation. This study offers a viable new approach for 3D‐bioprinting for the construction of bone substitutes in tissue regeneration.

## 5 Experimental Section

*Materials*: PEG (*M*_w_ = 4K and 10K, Sigma‐Aldrich, St. Louis, USA), acryloyl chloride (98%, TCI, Shanghai, China), triethylamine (99%, TCI, Shanghai, China), diethyl ether (Lingfeng Chemical Reagent Company, Shanghai, China), 2‐hydroxy‐2‐methyl‐1‐phenyl‐1‐propanone (IRGACURE 1173, 98%, Sigma‐Aldrich, St. Louis, USA), Laponite XLG ([Mg_5.34_Li_0.66_Si_8_O_20_(OH)_4_]Na_0.66_; BKY, Wesel, Germany), and HA (*M*_w_ = 350K, TCI, Shanghai, China) were all used as received. All other chemicals and solvents were analytical reagents and were purchased from Lingfeng Chemical Reagent Company (Shanghai, China) and used as received.

*Cell Culture*: Primary ROBs were isolated from minced SD rats (born within 3 d) calvarial chips, as described previously;^26^ this procedure was conducted in accordance with the guidelines set by the Ethics Committee for Animal Research, Shenzhen Institutes of Advanced Technology, Chinese Academy of Sciences. ROBs, between the third to fifth passage, were used in the 3D‐bioprinting system to evaluate cell viability, proliferation, and differentiation ability both in vitro and in vivo. ROBs were cultured in α‐MEM (Hyclone, Utah, USA) with 10% (v/v) fetal bovine serum (Corning, New York, USA), supplemented with antibiotics (100 U mL^−1^ penicillin, 100 µg mL^−1^ streptomycin) and incubated at 37 °C with 5% carbon dioxide (CO_2_).

*Prehydrogel Solution Preparation for 3D‐Bioprinting*: Crosslinkable PEGDA of different molecular weights (*M*_w_ = 4K and 10K) were synthesized as previously described.^9^ The recipes for preparing prehydrogel solutions are listed in Table S1 in the Supporting Information. Briefly, PEGDA was dissolved in deionized water at first, and then 3 wt% of the photoinitiator IRGACURE 1173 (relative to the mass of PEGDA crosslinker) was added into the solution and stirred thoroughly under a nitrogen atmosphere until completely dissolved. The solution was then sterilized via filtration through a 0.22 µm filter. Subsequently, ultraviolet sterilized nanoclay (Laponite XLG) was added into the solution and vigorously stirred on a clean bench for about 2 h, to obtain a homogeneous prehydrogel solution. Bubbles were removed from the prehydrogel solution by centrifugation (3000 rpm, 10 min) before being used.

Sterilized HA powder (20%) was dissolved in sterile phosphate buffer solution (PBS, 100 mL) and stirred thoroughly on a clean bench until dissolved. For 3D‐bioprinting preparation, a cell suspension (50 µL) with 4 × 10^6^ ROBs cells was added into 20% HA (1.0 g) solution and gently stirred to achieve uniformity for printing.

*3D‐Bioprinting Process*: The printing device (3D scaffold printer) used for our experiments is a precision three‐axis positioning system (Bioscaffolder 3.1, GeSiM, Grosserkmannsdorf, Germany). The system was placed on a clean bench and was sterilized using 75% alcohol and UV light before use. In our study, a two‐channel printing method was used, one channel for supporting matrix printing (PEG–Clay) and another for cell printing (HA); the printing process is shown in Figure 1 and Movie S1 in the Supporting Information. The dosing pressure of the syringe pump for the PEG–Clay prehydrogel solution and the cell containing HA solution was controlled between 55 and 65 and 70 and 80 kPa, respectively. The moving speed of the dispensing unit was set to 15 mm s^−1^. The cell containing scaffolds were printed in 60 mm diameter cell culture dishes and placed in a crosslinking oven (XL‐1000 UV Crosslinker, Spectronics Corporation, NY, USA) for 10 min polymerization under UV. Then, α‐MEM medium (8 mL) was added into each dish. The medium was changed the next day to allow time for HA cell release and adhesion within the PEG–Clay scaffold.

Pure PEG–Clay scaffolds without ROBs, with the same structure as the 3D‐bioprinting scaffolds, were also printed by this system. Furthermore, PEG hydrogels and PEG–Clay hydrogels were prepared without printing, for basic characterization, and compression tests.

*Basic Characterizations*: PEG (15 mg) or pure PEGDA (15 mg) was dissolved in D_2_O (0.5 mL), and their respective ^1^H NMR spectra were recorded on an AVANCE III 400 spectrometer (BRUKER, Madison, USA). FTIR spectroscopy (BRUKER VERTEX 70, Madison, USA), and TGA (Q600 SDT, TA Instruments, New Castle, USA) were used to characterize the nanoclay, PEGDA, PEG hydrogels, and PEG–Clay nanocomposite hydrogels. TGA was performed from room temperature to 1090 °C, at a heating rate of 10 °C min^−1^ in nitrogen.^27^ The microstructure of the PEG–Clay scaffolds was investigated by TEM (Philips CM100, Massachusetts, USA).^28^ For TEM imaging, lyophilized PEG–Clay scaffolds were embedded in epoxy resin for microtoming at −40 °C with a glass knife to ≈50 nm thick sections, which were then deposited on copper grids for imaging at 200 kV.

*Compression Test*: To assess if the incorporation of the clay component into the hydrogel system improved the mechanical properties of the hydrogels, the compression properties of the PEG hydrogels and PEG–Clay hydrogels were tested on an Instron 5697 (Instron, Grove City, USA) universal material testing system at room temperature. All hydrogels were tested directly after polymerization. As pure PEG hydrogels are unprintable, all samples used in the compression test were prepared in a cylindrical shape, 4 mm in diameter, and 4 mm in height. The crosshead speed was set to 10 mm min^−1^. At least five samples were used for each test for statistical significance.

*Ion Leaching Analysis*: Immersion tests were carried over different time points (from 1 to 21 d) to assess the quantities of magnesium and silicon ions released from the 3D‐printed pure PEG–Clay scaffolds with different PEGDA molecular weights. Printed scaffolds, 15 mm in width and five layers high, were immersed in PBS (8 mL) separately. Nine different periods of time (1, 2, 3, 4, 5, 6, 7, 14, and 21 d) were analyzed by inductively coupled plasma optical emission spectrometry (Perkin Elmer, Optima 7000DV, Massachusetts, USA) to determine the concentration levels of Mg^2+^ and Si^4+^ released and establish the relationship between ion release and time.

*Rheology Test*: Dynamic rheological experiments with 20%PEG4K–Clay prehydrogel solutions of different nanoclay concentrations and 20% HA (20 g HA powder dissolved in 100 mL PBS) were carried out using a rheometer (MCR302, Anton Paar, Austria). Plate‐plate geometry with a plate diameter of 25 mm was used. The shear rates were controlled between 1 and 100 s^−1^ at room temperature.

*Proliferation Assay and Cell Morphology*: Cell proliferation ability was determined after 1, 3, 5, and, 7 d of culture through the CCK‐8 assay, after 3D‐bioprinting.^29^ After incubation with 10% CCK‐8 solution at 37 °C for 4 h, cell proliferation was quantified by measuring the optical density of the CCK‐8 solution at 450 nm, using a Multiskan spectrum reader (Bio Tek Synergy4, Winooski, USA). For testing, each 15 × 15 mm^2^ printed scaffold was equally divided into four parts both for the proliferation and differentiation assays and the culture medium was 2 mL per part. In order to verify the advantages of the 3D‐bioprinting method over the traditional cell‐seeding method, the ROBs were seeded onto the top of the sterilized PEG–Clay scaffolds (7.5 × 7.5 × 2–7.5 × 7.5 × 3 mm^3^) placed in 24‐well cell culture plates (Corning, New York, USA). The same cell density was used for cell seeding as the cell‐printing method, and the culture conditions were the same.

The live/dead viability assay^30^ was performed according to the manufacturer's instructions to test the viability of ROBs one day after printing and after 7 d culture. The samples were observed through laser scanning confocal microscopy (Leica SD AF, Hamburg, Germany). Excitation of 488 nm was used to detect the live (green) cells stained by calcein AM, and 561 nm excitation was used to observe dead (red) cells stained by EthD‐1.

For cell morphology observations after 3D‐bioprinting and 7 d culture, ROBs cytoskeleton was stained with 50 µg mL^−1^ phalloidin‐rhodamine (Sigma‐Aldrich, St. Louis, USA) after fixation with 4% paraformaldehyde (PFA) and permeation by 0.1% Triton.^31^ SEM (Hitachi S4800 FEG, Tokyo, Japan) was used to detect the growth status of the ROBs within the PEG4K–Clay scaffolds after 3D‐bioprinting, fixation, gradient dehydration, and critical point drying.

*Differentiation Assay*: The quantification of ALP activity and ALP staining were used to evaluate the osteoblast phenotype of ROBs grown within the scaffolds after 3D‐bioprinting.^32^ After 4, 7, 14, and 21 d culture, the scaffolds printed with ROBs were rinsed three times with PBS, and then lysed in lysis buffer (300 µL, radio immunoprecipitation assay (RIPA) buffer, Beyotime Biotechnology, Shanghai, China). Cell debris was removed by centrifugation at 13 rpm, at 4 °C for 5 min, and then the supernatant (50 µL) was added to chromogenic substrate (50 µL) in a 96‐well plate and incubated at 37 °C for 2 h. Then, stop buffer (100 µL) was added to stop the reaction. Absorbance was measured at 405 nm using a microplate reader. Analysis of each sample was performed in triplicate and the total protein content was used to normalize the ALP activity by a commercially available protein assay kit (Pierce TM BCA Protein Assay Kit, ThermoFisher Scientific, Massachusetts, USA). In order to visualize the differentiation ability of ROBs printed on each scaffold, ALP staining for each scaffold was performed using a 5‐bromo‐4‐chloro‐3‐indolyl phosphate (BCIP)/nitrobluetetrazolium chloride (NBT) Alkaline Phosphatase Color Development Kit (Beyotime Biotechnology, Shanghai, China).

*In Vivo Evaluation*: All the animal procedures and experiments were approved by the Ethics Committee for Animal Research, Shenzhen Institutes of Advanced Technology, Chinese Academy of Sciences. A rat tibia model was used for the bone defect repair experiment and the surgical procedures were all conducted under sterile conditions. The scaffolds both printed with ROBs and without ROBs were cut into sections of 2.5 mm diameter and 5 mm length. These sections were implanted into tibia bone defects of 12‐week old male SD rats. First, the rats were anesthetized with pentobarbital sodium (40 mg kg^−1^) by intraperitoneal injection. Then, the critical size defects (2.5 mm diameter and 5 mm length) were created close to the tibia plateau and in the middle of the tibia shaft of an SD rat leg by a 2.5 mm drill. The implants were placed bilaterally resulting in two implants per rat, and the wound was closed carefully. Twelve SD rats were randomly divided into three groups corresponding to 3D‐bioprinting PEG4K–Clay scaffolds, pure PEG4K–Clay scaffolds without ROBs, and blanks as the controls. All rats were sacrificed 8 weeks after implantation, and the implants were harvested and fixed in 4% PFA for the micro‐CT assay and histological analysis later.

In order to characterize the new bone formation and mineralization, a polychrome sequential fluorescent labeling method was used.^21^ Three different fluorochromes were sequentially administered intramuscularly, 25 mg kg^−1^ tetracycline hydrochloride, 30 mL kg^−1^ alizarin red S, and 20 mg kg^−1^ calcein 2, 4, and 6 weeks after the operation.

For the ectopic osteoinduction experiment, 24 SD rats (12‐weeks old) were used. Following disinfection of the operative region, the 3D‐bioprinting PEG4K–Clay scaffolds (using GFP‐labeled ROBs) were implanted into muscle pouches created in the left thigh of each rat. The control group pure PEG4K–Clay scaffolds without ROBs were implanted into the muscle pouches in the right thigh of each rat. The wounds were sutured carefully. At every time point (1, 3, 5, 7, 14, and 21 d, 4, and 8 weeks after implantation), three rats were euthanized. For the first 1–21 d time points, cell‐laden PEG4K–Clay scaffolds were exfoliated from the tissues to confirm the cell viability after implantation. For the 2, 4, and 8 week time points, the implanted region including the surrounding tissues was excised. The excised fragments were fixed in 4% PFA for histological analysis and the 8 week old implant scaffolds were analyzed by micro‐CT.

New bone formation was determined by micro‐CT (SkyScan 1176, Bruker, Madison, USA) for the fixed samples. The scanning parameters were set at 60 kV, Al 1 mm filter, and 18 µm resolution. After scanning, the NRecon software (Skyscan, USA) and CTvol program (SkyScan) were used to reconstruct 2D and 3D models of the samples, and DataViewer software (SkyScan) and CTAn program (SkyScan) were used to determine the bone volume around the implants.

After micro‐CT, 50 µm undecalcified sections were prepared using a Exakt system (model 310 CP bnad system, Exakt, Oklahoma City, OK, USA) for fluorescence labeling observation under a confocal laser scanning microscope (Leica TCS SP8, Hamburg, Germany). The excitation/emission wavelengths used to observe the chelating fluorochromes were 405/575, 543/620, and 488/520 nm for tetracycline hydrochloride (yellow), alizarin red S (red), and calcein (green), respectively. After fluorescence microscopy, Giemsa staining was used to visualize the mineralized bone tissue (pink) in the same sections.^18^ The images were captured by a fluorescence microscope (Olympus, BX53, Tokyo, Japan).

For paraffin sections, all samples (both tibias and excised fragments) were decalcified in 10% ethylene diamine tetraacetic acid (EDTA) for 6 weeks after fixation, embedded in paraffin and sectioned into 5–7 µm sections (Leica RM2235, Hamburg, Germany). Hematoxylin (Sigma‐Aldrich, St. Louis, USA) and eosin (C0105, Beyotime Biotechnology, Shanghai, China) (H&E) staining^22^ and Goldner's staining^24^ were used to detect the specific tissue response to the implanted materials. Images were taken under the fluorescence microscope (Olympus, BX53, Tokyo, Japan).

*Statistical Analysis*: All the experiments were analyzed by one‐way analysis of variance (ANOVA) with Tukey' post hoc test and expressed as means ± standard deviations (SD). A *p*‐value < 0.05 was considered to be statistically significant.

## Conflict of Interest

The authors declare no conflict of interest.

## Supporting information

Supplementary

Supplementary
