# Supplementary material for: 3D‐Bioprinted Osteoblast‐Laden Nanocomposite Hydrogel Constructs with Induced Microenvironments Promote Cell Viability, Differentiation, and Osteogenesis both In Vitro and In Vivo
Source: Adv Sci (Weinh). 2017 Nov 24;5(3):1700550. doi: 10.1002/advs.201700550 (PMC5867050; doi:10.1002/advs.201700550)
Supplement: Supplementary file 1 — Supplementary [file ADVS-5-1700550-s001.pdf]

Copyright WILEY-VCH Verlag GmbH & Co. KGaA, 69469 Weinheim, Germany, 2017.

ADVANCED SCIENCE Open Access

Supporting Information

for *Adv. Sci.,* DOI: 10.1002/advs.201700550

3D-Bioprinted Osteoblast-Laden Nanocomposite Hydrogel Constructs with Induced Microenvironments Promote Cell Viability, Differentiation, and Osteogenesis both In Vitro and In Vivo

Xinyun Zhai, Changshun Ruan, Yufei Ma, Delin Cheng, Mingming Wu, Wenguang Liu, Xiaoli Zhao, Haobo Pan,* and William Weijia Lu**

Supporting Information

3D-bioprinted Osteoblast-laden Nanocomposite Hydrogel Constructs with Induced Microenvironments Promote Cell Viability, Differentiation and Osteogenesis both *In Vitro* and *In Vivo*

Xinyun Zhai, Changshun Ruan, Yufei Ma, Delin Cheng, Mingming Wu, Wenguang Liu, Xiaoli Zhao, Haobo Pan*, William Weijia Lu**

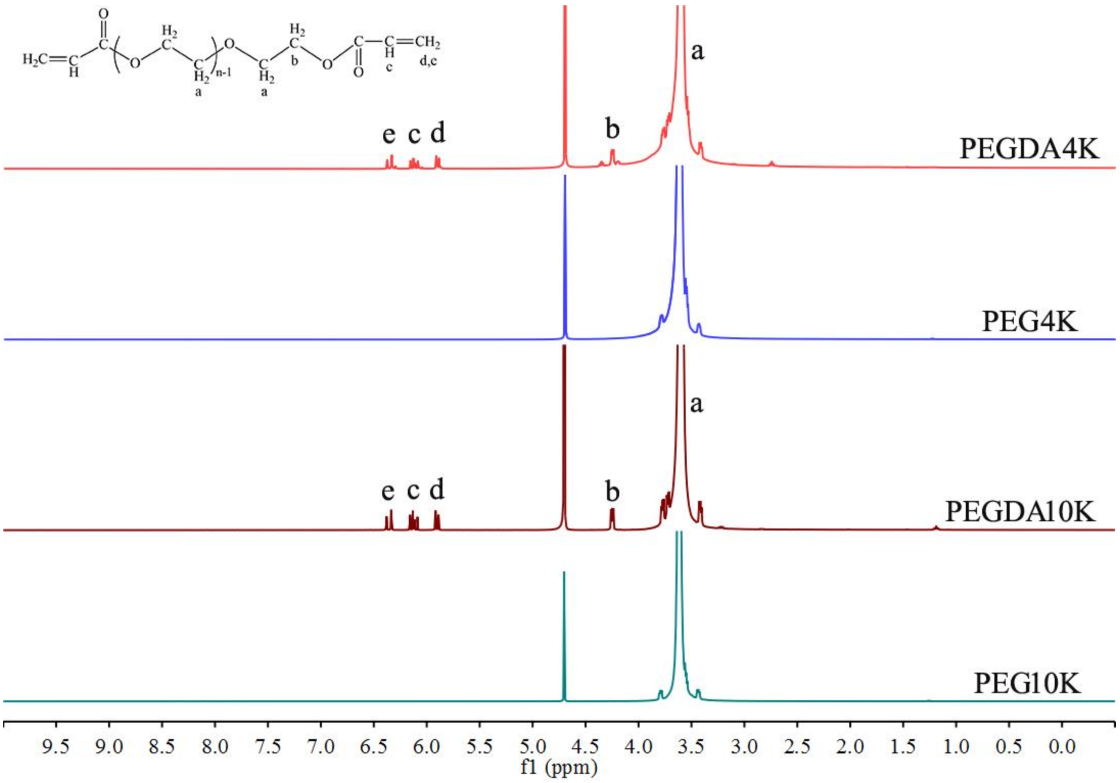

Figure S1. ^{1}H NMR spectra of PEG4K, PEG10K, PEGDA4K and PEGDA10K.

^{1}H NMR (D_2O): δ=6.35 (d, 2H, *cis* CH_2=CH), δ=5.80 (d, 2H, *trans* CH_2=CH), δ=6.10 (m, 2H, ROOCC*H*=CH_2), δ=4.25 (t, 4H, CH_2=CHCOOCH_2-CH_2O-), δ=3.5–3.6 (m, 360H, -CH_2CH_2O-).[1]

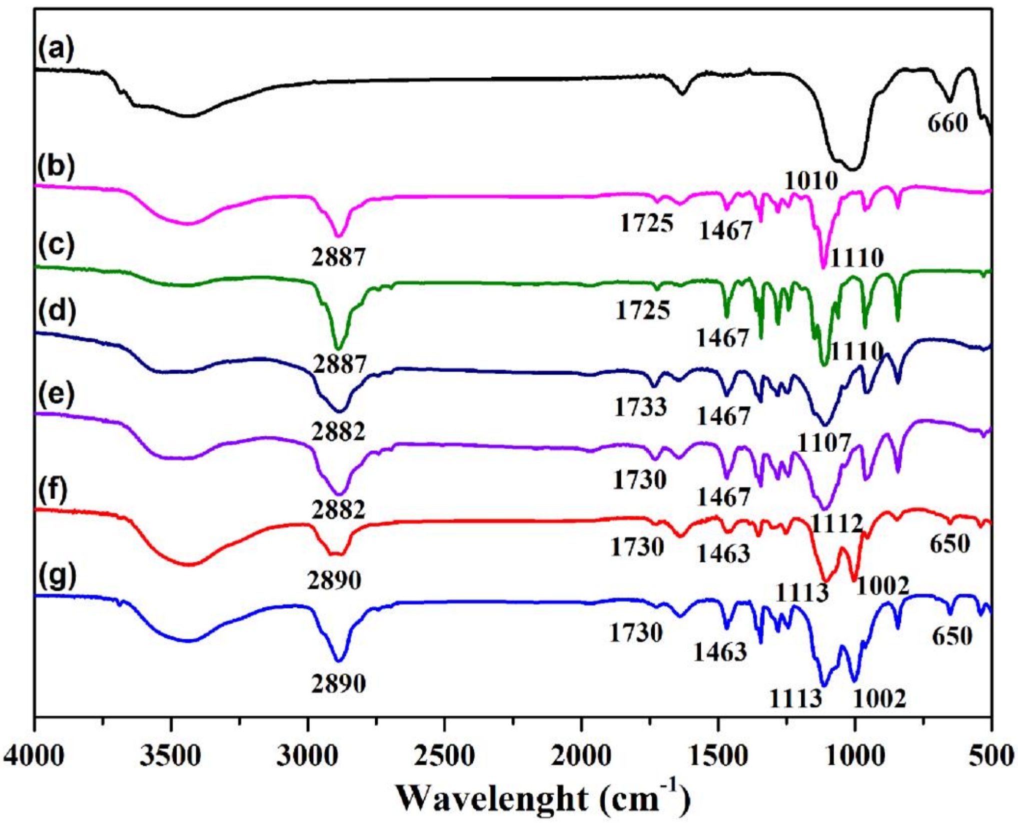

Figure S2. FTIR spectra of nanoclay (a), PEGDA4K (b), PEGDA10K (c), PEG4K hydrogel (d), PEG10K hydrogel (e), 20%PEG4K-7%Clay hydrogel (f) and 20%PEG10K-7%Clay hydrogel (g).

As shown in the figure, besides Si-O stretching and bending bands appearing at 1002 cm^{-1} and 650 cm^{-1}, PEG-Clay hydrogel also shows C-H stretching bands at 2890 cm^{-1}, C=O stretching vibration at 1730 cm^{-1}, C-O-C stretching bands at 1113 cm^{-1} and $-CH_2-$ bending vibration at 1463 cm^{-1}, which can also be found out in PEGDA crosslinker and pure PEG hydrogel. This suggests the formation of hybrid hydrogel.[1, 2]

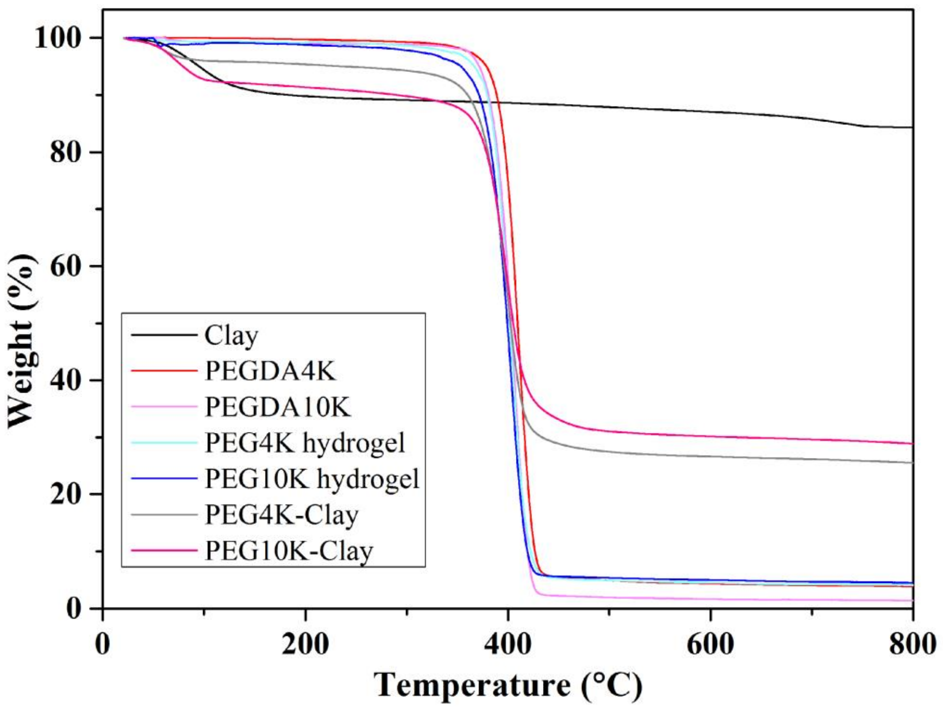

Figure S3. TGA curves of nanoclay, PEGDA4K, PEGDA10K, PEG4K hydrogel, PEG10K hydrogel, 20%PEG4K-7%Clay hydrogel and 20%PEG10K-7%Clay hydrogel.

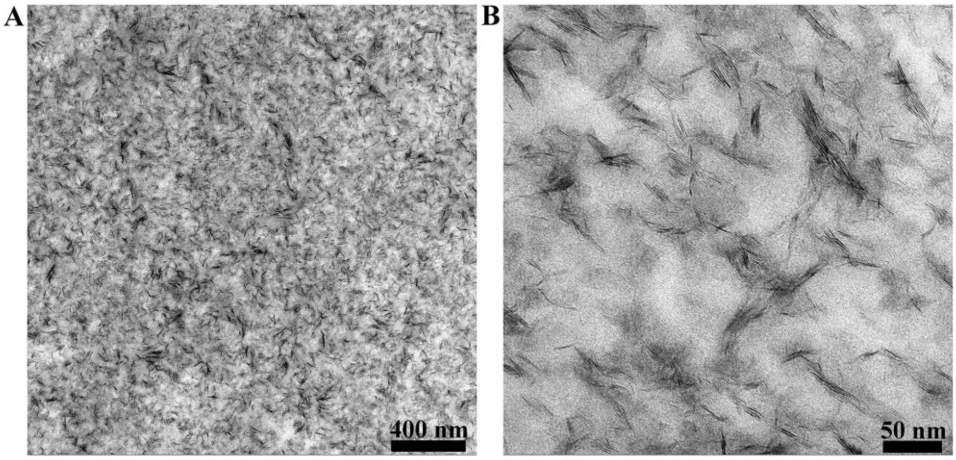

Figure S4. TEM images showing the exfoliation and dispersion of clay in the PEG4K-Clay hydrogel, where the dark platelets represent the exfoliated clay.

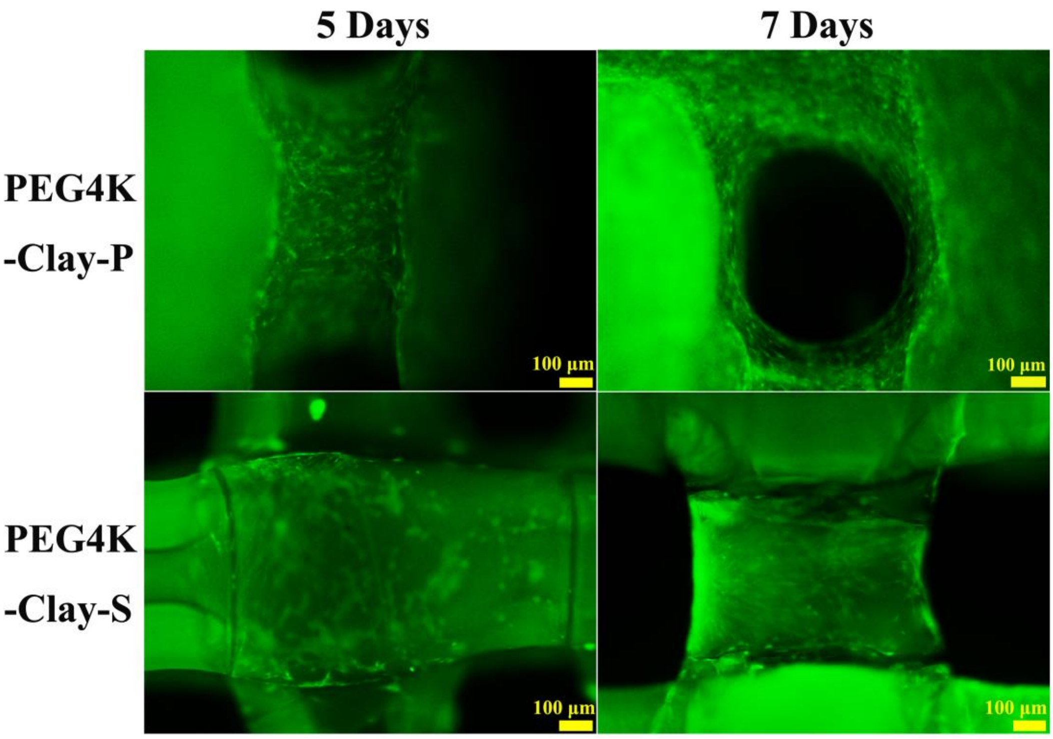

Figure S5. The status of ROBs after 3D-bioprinting (PEG4K-Clay-P) or traditional seeding (PEG4K-Clay-S) on PEG4K-Clay scaffolds after culturing for 5 and 7 days under 100× magnification.

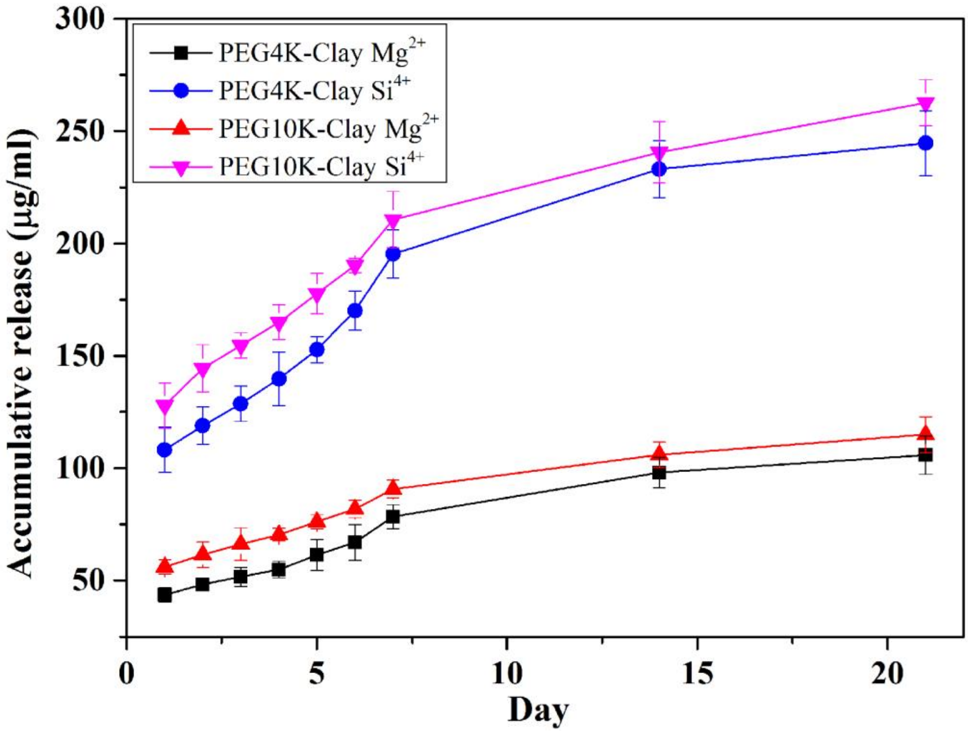

Figure S6. Accumulative release of magnesium ions and silicon ions from PEG-Clay scaffolds as a function of time.

Table S1. Preparation of PEG-Clay and PEG pre-hydrogel solutions with varied monomer concentrations.

Sample	PEGDA (g)	Laponite XLG (g)	H_2O (μL)
20%PEG4K-3%Clay	0.20	0.03	1000
20%PEG4K-5%Clay	0.20	0.05	1000
20%PEG4K-7%Clay	0.20	0.07	1000
20$PEG4K-10%Clay	0.20	0.10	1000
20%PEG10K-3%Clay	0.20	0.03	1000
20%PEG10K-5%Clay	0.20	0.05	1000
20%PEG10K-7%Clay	0.20	0.07	1000
20%PEG10K-10%Clay	0.20	0.10	1000
PEG4K	0.20	0	1000
PEG10K	0.20	0	1000

References

[1] J. Zhang, N. Wang, W. Liu, X. Zhao, W. Lu, *Soft Matter* **2013**, *9*, 6331

[2] H. Li, R. Wu, J. Zhu, P. Guo, W. Ren, S. Xu, J. Wang, *J. Polym. Sci., Part B: Polym. Phys.* **2015**, *53*, 876.
